# Deterministic abiotic filtering and halophilic core microbiomes shape bacterial community assembly in coastal salt flats (sabkha) of southern Morocco

**DOI:** 10.1128/aem.00061-26

**Published:** 2026-05-11

**Authors:** Ghita Amechatte, Nabil Radouane, Ayoub El Mouttaqi, Danilo Licastro, Abdelaziz Hirich, Hijri Mohamed, Bulbul Ahmed

**Affiliations:** 1 African Genome Center (AGC), University Mohamed VI Polytechnic (UM6P) 479571, Benguerir, Morocco; 2 African Sustainable Agriculture Research Institute (ASARI), College of Agriculture & Environmental Sciences (CAES), Mohammed VI Polytechnic University (UM6P) 479571, Laayoune, Morocco; 3 Area Science Park 18469, Trieste, Italy; 4 Institut de Recherche en Biologie Végétale (IRBV), Département de Sciences Biologiques, Université de Montréal 467891 , Montréal, Quebec, Canada; Colorado School of Mines, Golden, Colorado, USA

**Keywords:** abiotic filtering, halophytes, hypersaline ecosystems, halophilic bacteria, sabkha (coastal salt flats)

## Abstract

Coastal salt flats, locally known as sabkhas, are hypersaline, alkaline desert ecosystems that impose extreme abiotic stress on microbial and plant life. Despite their ecological significance, plant-associated microbiomes in these habitats remain poorly characterized. In this study, we investigated the bacterial communities of native halophytes across three sabkha sites in southern Morocco using an integrated culture-independent and culture-dependent framework. Soil physicochemical analyses revealed strong gradients in salinity and ionic composition, along with consistent alkaline pH across sites. These conditions strongly structured bacterial assemblage: alpha diversity declined progressively from bulk soil to rhizosphere soil, root, and shoot; and beta diversity showed clear compartmental separation driven by environmental factors. Canonical correspondence analysis identified electrical conductivity (EC), Na₂O, K₂O, and carbonate fractions as the main abiotic drivers. Across plant species, bacterial communities converged toward a stable halophilic core microbiome dominated by *Halomonas*, *Kushneria,* and *Marinococcus*, with 66% of amplicon sequencing variants (ASVs) shared across compartments. Host identity played a secondary role as environmental filtering overshadowed host-specific associations. Culture-dependent isolation recovered 19 halophilic and halotolerant bacterial strains, including representatives of *Halomonas*, *Idiomarina*, *Marinobacter*, *Psychrobacter*, *Planomicrobium,* and *Bacillus*. These isolates exhibited robust growth on saline Marine Agar medium, indicating strong salt tolerance consistent with their occurrence in hypersaline environments. The strong concordance between cultured isolates and metabarcoding profile confirms that dominant halophilic lineages are both ecologically robust and readily culturable. Together, these findings demonstrate that sabkha plant microbiomes are primarily shaped by deterministic abiotic filtering and harbor resilient, stress-adapted bacterial communities. Sabkhas thus represent promising reservoirs of halophilic microbes with potential applications in saline agriculture and improving crop resilience under extreme environmental conditions.

**IMPORTANCE:**

Coastal salt flats (sabkhas) are among the most extreme terrestrial environments, characterized by high salinity, alkalinity, and limited water availability. As soil salinization expands worldwide, understanding how life persists in such habitats is increasingly important for sustainable agriculture. This study shows that sabkha ecosystems impose strong environmental filtering on plant-associated bacterial communities, leading to highly structured microbiomes across soil, root, and shoot compartments. Despite differences among sites and plant species, bacterial communities converged toward a shared halophilic core microbiome, dominated by salt-adapted genera that are resilient to extreme ionic stress. Importantly, many of these dominant bacteria were readily culturable, highlighting sabkhas as accessible reservoirs of stress-tolerant microbes. Our findings demonstrate that abiotic conditions outweigh plant identity in shaping microbiome assembly under extreme stress and reveal sabkha halophytes as valuable natural models for discovering microbes with potential applications in saline agriculture, soil restoration, and crop resilience in salt-affected environments.

## INTRODUCTION

Sabkha ecosystems provide a compelling natural context for studying microbial adaptation to extreme abiotic stress. The term sabkha, derived from the Arabic word for “salt flat,” refers to hypersaline landscapes distributed across North Africa, the Arabian Peninsula, and parts of Australia. Both coastal and inland sabkhas experience extreme salinity, high temperatures, low water availability, and intense evaporation, resulting in soils with elevated pH, high carbonate content, and distinctive ionic profiles (1). Similar environmental constraints have been shown to shape the root-associated microbiomes of plants in Sahara desert in southern Morocco, where both soil physicochemical properties and plant identity influence bacterial community composition (2). Despite these harsh abiotic conditions, sabkhas host specialized halophytic vegetation whose associated microbiomes remain largely unexplored.

Increasing evidence highlights the central role of environmental context in shaping plant-microbe interactions. Environmental factors, such as soil nutrients, pH, temperature, salinity, and water availability, are major determinants of the composition and function of plant-associated microbial communities (3–6). In benign and moderate environments, plants often exert strong control over microbiome assembly through root exudates, immune signaling, and developmental cues, enabling them to recruit beneficial microbes and structure highly diverse, host-specific communities (6, 7). In contrast, in extreme ecosystems, such as saline soils, arid regions, or sabkhas, abiotic constraints including high salinity, drought, or elevated temperatures frequently override host-driven selection, limiting microbial diversity and narrowing microbial plasticity. These stressful conditions often lead to the convergence of taxonomically unrelated plants toward functionally similar or stress-tolerant microbial communities enriched in halophilic or extremotolerant taxa (5, 8–10). Transitional or disturbed environments may show intermediate patterns in which both host and environmental drivers operate in context-dependent ways, producing dynamic shifts in microbial richness, functional potential, and community resilience (11–13).

These contrasting mechanisms have important implications for climate change and agricultural adaptation. In harsh or rapidly changing environments, strong environmental filtering can reduce the plant’s ability to shape its microbiome; however, the persistence of functionally redundant, stress-tolerant microbial groups may still confer essential support for plant survival (4, 8, 14). Leveraging beneficial microbes, including halotolerant bacteria and stress-adapted mycorrhizal fungi, represents a promising strategy to enhance crop performance in degraded or saline soils, provided that microbial traits are well matched to local abiotic conditions (2, 10, 14, 15). Soil salinity imposes severe stress on plants but can be mitigated through inoculation with salt-tolerant rhizobacteria that improve plant salt resistance and growth in saline soils. Halotolerant plant growth-promoting rhizobacteria have been shown to help plants tolerate salinity stress and support sustainable agriculture in salt-affected soils. Therefore, understanding how host genetics interacts with the environmental context is fundamental for predicting microbiome assembly under climate change and for designing effective microbial-based interventions (6, 16, 17). Collectively, these insights reinforce that environmental context is a primary determinant of plant-microbe interactions, particularly under extreme abiotic stress where environmental filtering can dominate over host control.

Plants coexist with diverse microbial communities that colonize both external and internal tissues, forming complex holobionts essential for plant function and fitness. Among these microbial partners, bacteria are particularly influential due to their abundance, metabolic versatility, and capacity to form intimate associations with a wide range of plant hosts. The assembly of plant-associated microbiomes is shaped by a combination of host-derived factors, including genotype, root exudation patterns, developmental stage, and immune activity, and environmental factors (18, 19). Life-history traits also influence microbial recruitment; for example, perennial plants often harbor richer and more phylogenetically diverse microbiomes than annuals, owing to their longer lifespans, deeper root systems, and more stable rhizosphere environments (20). Yet, under extreme abiotic stress, such as high salinity, drought, alkalinity, or heat, soil physicochemical properties frequently emerge as the dominant determinants of microbial community structure (21). Across these ecosystems, taxonomically unrelated plants may converge on similar microbiomes enriched in stress-tolerant bacterial lineages such as *Halomonas*, *Marinobacter*, *Salinimicrobium,* and *Pseudomonas,* reflecting environmental determinism rather than host specificity (22). These patterns underscore the need to disentangle the relative contributions of host-driven and environment-driven selection in microbiome assembly.

In this study, we investigated the structure and assembly of microbiomes associated with native halophytes at three sites within the coastal Oum Dbaa sabkhas in southern Morocco. Using integrated culture-independent and culture-dependent approaches, we aimed to determine whether microbiome assembly in these extreme habitats is governed predominantly by abiotic filtering or by host-specific selection. We hypothesized that (i) strong edaphic constraints, particularly salinity, ionic composition, and alkalinity, act as the primary drivers of bacterial community structure, overriding host genotype effects; (ii) bacterial communities converge toward a shared halophilic core microbiome across different plant species, indicative of deterministic environmental filtering; and (iii) the dominant halophilic taxa detected through metabarcoding will also be recoverable through cultivation, reflecting their ecological robustness and applied potential. By combining community profiling with culturable strain isolation, this study provides a comprehensive mapping of bacterial communities in sabkha ecosystems and identifies promising halophilic and halotolerant candidates for developing stress-resilient microbial inoculants to support sustainable agriculture under extreme environmental conditions.

## MATERIALS AND METHODS

### Study site and sampling

**Fig 1:**
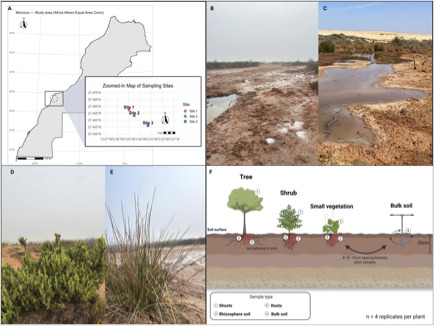
Study areas, sampling strategy, and sabkha landscape. (**A**) Map showing the three sampling sites within sabkha ecosystems of southern Morocco. The inset shows a zoomed view of the spatial distribution of the three sites. The map was generated in R (v4.3.1) using the packages ggplot2, sf, and rnaturalearth; underlying geographic data were obtained from the Natural Earth data set. (**B and C**) Field of the sabkha environments. (**D and E**) Dominant vegetation types sampled in this study, including examples of typical saline terrain and halophytic plant species. (**F**) Schematic illustration of the sampling design across plant functional groups: trees, shrubs, and small vegetation. For each plant, four compartments were sampled: shoot (1), root (2), rhizosphere soil (3), and bulk soil (4), with ≥20–30 cm spacing between replicates (*n* = 4 per plant type).

Sampling was conducted at three sites within the Oum Dbaa coastal sabkha ecosystem in southern Morocco (Fig. 1A). The study sites were located at 27°27′27″N, 13°02′59″W; 27°27′12″N, 13°02′42″W; and 27°26′46″N, 13°01′58″W. The geographical map of sampling locations was generated in R (v4.3.1) using the packages ggplot (23), sf (24), and rnaturalearth (25), based on the recorded GPS coordinates of each site. At each sabkha, representative halophytic plant species were sampled for microbiome analysis (see Table S1 at https://doi.org/10.6084/m9.figshare.32002416). A total of ten halophytes were included (Fig. 1C and D), encompassing shrubs [*Arthrocaulon macrostachyum* (Moric.) Piirainen & G. Kadereit and *Halocnemum strobilaceum* (Pall.) M. Bieb], small trees [*Tamarix amplexicaulis* Ehrenb. and *Nitraria retusa* (Forssk.) Asch.], grasses (*Juncus acutus* L.), and perennial herbs [*Saharanthus ifniensis* (Caball.) M. B. Crespo & Lledó, *Caroxylon tetrandrum* (Forssk.) Akhani & Roalson, *Caroxylon tetragonum* (Dalile) Moq., *Tetraena gaetula* (Emb. & Maire) Beier & Thulin., and *Frankenia corymbosa* Desf.]. For each plant species, four biological replicates were collected, and from each replicate, three compartments were sampled: rhizosphere soil, root, and shoot tissues (Fig. 1B). At each site, three bulk soil samples (0–20 cm depth) were collected away from plant roots to serve as environmental controls and for soil physicochemical properties. In total, 151 samples were obtained, including 142 plant-associated samples and nine bulk soil samples. Immediately after collection, all samples were stored in a refrigerator at ~4°C in the mobile field laboratory vehicle (African Sustainable Agriculture Research Institute: ASARI, Laayoune, Morocco) and then transported and stored at –20°C prior to DNA extraction and isolation of bacteria.

### Soil physicochemical analysis

Soil physicochemical characterization was carried out at the ASARI (Laayoune, Morocco). Each soil replicate was analyzed individually across all parameters, without homogenization, to reflect natural site-level variability. Soil pH was measured in a 1:2.5 soil-to-water suspension following ISO 10390:2005. Electrical conductivity (EC) was determined in the saturated paste extracts according to NF ISO 11265, and soil salinity was inferred from EC values. Total carbonate content was quantified by the ISO 10693 (1995) volumetric method and active carbonate by NF X 31-106. Available phosphorus (P₂O₅) was measured using the Olsen extraction method (NF ISO 11263). Organic carbon (OC) was analyzed following NF ISO 14235 and converted to organic matter (OM) using OM = OC × 1.724. Total nitrogen (N) was measured using the Kjeldahl digestion method. For elemental composition, soil samples were digested with concentrated sulfuric acid, and macroelements (K₂O, CaO, Na₂O, and MgO) and microelements (Fe, Zn, Cu, and Mn) were quantified by atomic absorption spectroscopy (AAS).

### Bacterial isolation

Bacteria isolation from the root, shoot, and rhizosphere samples was performed following the protocol of reference 26 using Marine Agar (Difco 2216; BD Difco, Franklin Lakes, NJ, USA), a saline medium formulated for halotolerant microorganisms and containing elevated NaCl concentrations. For epiphytic bacteria, plant tissues were rinsed three times with sterile distilled water to remove loosely attached debris, cut into ~1 cm fragments, and then rinsed three times with sterile distilled water to remove loosely attached particles. The surface-associated microbial fraction was directly plated onto Marine Agar under sterile conditions. For endophytic bacteria, plant tissues were surface-sterilized using 70% ethanol for approximately 1 min, followed by three successive rinses with sterile distilled water to remove residual ethanol. After sterilization, tissues were aseptically sectioned, the outer surface was removed, and internal fragments were directly placed onto Marine Agar plates for endophyte isolation. For rhizosphere samples, approximately 1 g of soil was suspended in 9 mL of sterile saline solution and shaken at 150 rpm for 45 min to detach microbial cells (27). The suspension was serially diluted (10^−^¹−10^−^⁶), and pilot tests determined the optimal dilution for plating. Aliquots (100 μL) were spread onto Marine Agar plates. Plates were incubated at 28°C. Colony emergence generally occurred after 48–72 hours, though some isolates required 96 to 120 h. Distinct colonies were repeatedly restreaked to obtain pure isolates. Isolates were considered salt-tolerant based on their ability to grow on Marine Agar under these saline conditions. A single colony from each isolate was subjected to direct colony PCR for taxonomic identification. One colony was transferred into 10 µL sterile nuclease-free water, macerated, and heated at 98°C for 10 min. The resulting lysate served as the PCR template. Amplification of the 16S rRNA gene used primers 27F (AGAGTTTGATCCTGGCTCAG) and 1492R (TACGGHTACCTTGTTCGACTT). PCR reactions (50 µL) contained 2 µL of each primer (10 µM), 10 µL GC enhancer reagent, 25 µL Master Mix (New England Biolabs, Ipswich, MA, USA), 1 µL of the colony lysate, and nuclease-free water. Cycling conditions were as follows: 95°C for 5 min; 35 cycles of 95°C for 30 s, 57°C for 60 s, and 72°C for 60 s, followed by final extension at 72°C for 10 min. Amplicons were visualized on 1% agarose gels using an iBright imaging system (Thermo Fisher Scientific, Waltham, MA, USA). Successful PCR products were purified and subjected to Sanger sequencing at Genome Quebec (Montréal, Canada). Forward and reverse sequences were assembled, quality-trimmed, and merged into contig using Geneious Prime v2025.2.1 (Biomatters, Auckland, New Zealand). Taxonomic identification was performed using BLASTn against the NCBI nucleotide database; only hits with ≥97% sequence identity and ≥98% coverage were accepted for genus- or species-level classification.

### DNA extraction, library preparation, and sequencing

Total genomic DNA was extracted using previously established methods (28) from 250 mg of the rhizosphere and bulk soil using the DNeasy PowerSoil Pro Kit (Qiagen, Toronto, ON, Canada) and from 100 mg of ground root tissue using the DNeasy Plant Mini Kit (Qiagen, Toronto, Canada), following the manufacturer’s instructions. Prior to homogenization, plant samples were flash-frozen in liquid nitrogen, while soil samples were processed directly. Both soil and plant samples were homogenized using a TissueLyser II (Qiagen, Hilden, Germany) with 2 mm tungsten beads at 24 Hz for 15 min. DNA was eluted in 50 µL of elution buffer for soil samples and in 20 µL for plant samples and stored at −20°C until further use. Extracted DNA concentration and purity were assessed using a BioSpectrometer (Eppendorf, Hamburg, Germany) and a NanoDrop ONE spectrophotometer (Thermo Scientific, Wilmington, DE, USA), and DNA integrity was verified by electrophoresis on 1% agarose gels stained with GelRed (1/10,000) and visualized using the GelDoc system (Bio-Rad, Montreal, QC, Canada). For bacterial community profiling, the V3–V4 region of the 16S rRNA gene was amplified with primers CS1_341 (5′-ACACTGACGACATGGTTCTACACCTACGGGNGGCWGCAG-3′) and CS2_806R (5′-TACGGTAGCAGAGACTTGGTCTGACTACHVGGGTATCTAATCC-3′). Libraries were prepared using the TruSeq Nano DNA LT Sample Preparation Kit (Illumina, United States) and sequenced on the Illumina NovaSeq 6000 platform with 2 × 150 bp paired-end reads at the Next-Generation Sequencing facilities of Laboratory of Genomics and Epigenomics (LAGE) in Area Science Park, Trieste, Italy. To monitor potential laboratory contamination, DNA extraction blanks and PCR-negative controls (nuclease-free water) were included and handled beside samples.

### Bioinformatic analysis of 16S rRNA sequences

Raw paired-end NovaSeq reads were processed in R v4.0.0 using the DADA2 package v1.18.0 (29). Quality filtering discarded reads containing ambiguous bases and those with more than three expected errors (maxEE = 3). No read truncation was applied, and PhiX spike-in contamination was removed. Denoising was performed with the DADA algorithm, followed by merging of paired reads and removal of chimeric sequences using the consensus method. Because NovaSeq quality scores are binned, which can lead to spurious sequence variants (30), only amplicon sequence variants (ASVs) present at least 250 times across the data set were retained for downstream analysis. Taxonomic assignment was carried out with the assign Taxonomy function in DADA2 against the SILVA database (release 138.2) (31) trimmed to the sequenced region. Nonbacterial sequences (chloroplast and mitochondrial) were removed prior to downstream analyses.

### Statistical analysis

To evaluate the effects of host identity and environmental variables on bacterial community structure, we conducted permutational multivariate analyses of variance (PERMANOVA) using the adonis2() function in the vegan R package (v2.6-4) (32). Bray-Curtis dissimilarities were computed from genus-level relative abundance data after prevalence filtering. Each factor was first tested independently in univariable PERMANOVAs using 999 permutations, and resulting *P*-values were adjusted for multiple testing with the Benjamini-Hochberg false discovery rate (FDR). To limit the influence of multicollinearity, variables with pairwise correlation coefficients |r| > 0.99 were excluded. The remaining predictors (*n* = 12) were included in a multivariable PERMANOVA, and the unique contribution of each variable was quantified by marginal R² values.

To correct for differences in sequencing depth, ASV counts were normalized to relative abundances using the “compositional” transformation implemented in the phyloseq package (33). Bacterial alpha diversity was quantified using the Shannon, Simpson, and Chao1 indices via the estimate_richness function in phyloseq. Group differences were tested with the Kruskal-Wallis test, followed by pairwise Wilcoxon rank-sum tests adjusted with Bonferroni correction. Boxplots were generated using ggplot2 (23), with statistical annotations added through ggpubr (34).

Beta diversity was assessed using Bray-Curtis dissimilarities derived from ASV-level relative abundances. Group-level differences were tested by PERMANOVA (adonis2(), vegan package in R), and the homogeneity of multivariate dispersions among groups was evaluated using the betadisper function in the same package. To identify microbial taxa differentiating plant compartments, we combined Random Forest classification and Indicator Species Analysis (IndVal.g). Random Forest models were trained using the randomForest R package (35) to rank genera by permutation importance, whereas IndVal.g (indicspecies package [36]) identified compartment-specific indicators based on permutation testing with FDR correction. These complementary approaches jointly revealed microbial genera that significantly contributed to the differentiation of bacterial communities of bulk soil, rhizosphere, root, and shoot. Detrended correspondence analysis (DCA) (37) was first applied to the ASV abundance matrix to assess the length of underlying ecological gradients and to guide the selection of an appropriate constrained ordination method. Gradient lengths were evaluated in standard deviation units along the DCA axes. Because the length of the first DCA axis exceeded four standard deviation units, indicating unimodal species responses, canonical correspondence analysis (CCA) (38) was subsequently used to examine relationships between bacterial community composition and environmental variables.

## RESULTS

### Soil physicochemical properties in the rhizosphere soils reflected bulk soil salinity gradients in sabkha

Comprehensive soil profiling across the three sabkha sites revealed pronounced heterogeneity in physicochemical conditions, with strong gradients in salinity and ionic composition (see Fig. S1 and Table S2 at https://doi.org/10.6084/m9.figshare.32002416). Site 1 exhibited the highest electrical conductivity (EC) in bulk soil, consistently exceeding 19,000 µS cm⁻¹, whereas Sites 2 and 3 showed substantially lower EC values (< 7,000 µS cm⁻¹) (Kruskal-Wallis, *P* < 0.05). Sodium oxide (Na₂O) and potassium oxide (K₂O) levels mirrored this pattern, reaching up to 51,916 mg kg⁻¹ Na₂O and 36,877 mg kg⁻¹ K₂O in Site 1. Despite differences in salinity, all soils maintained uniformly alkaline pH (8.4–9.2) and high carbonate content (18%–36% CaCO₃), reflecting the inherently harsh baseline conditions of sabkha ecosystems. To evaluate how these edaphic differences translate to the plant microenvironment, we analyzed physicochemical properties of rhizosphere soil (see Fig. S2 at https://doi.org/10.6084/m9.figshare.32002416). EC, Na₂O, and K₂O remained significantly higher in Site 1 compared with Sites 2 and 3 (Kruskal-Wallis with Dunn’s test, *P* < 0.05), confirming that plants in Site 1 experience extreme ionic stress, whereas plants in Site 3 are exposed to comparatively milder conditions.

### Alpha- and beta-diversity analyses reveal strong soil-plant filtering

Sequencing of the 16S rRNA gene data set yielded 6,010 bacterial ASVs across 151 samples. Taxonomic classification resolved 6,010 ASVs at the phylum level, 6,001 at the order level, and 2,316 at the genus level. Sequencing depth varied widely (9,820–671,482 reads per sample; mean = 228,014; median = 181,978). Rarefaction curves indicated sufficient sequencing coverage across all sample types (see Fig. S3 at https://doi.org/10.6084/m9.figshare.32002416).

**Fig 2:**
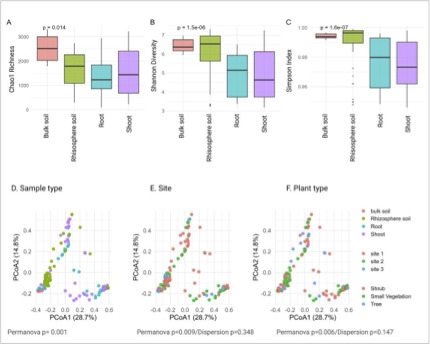
Alpha and beta diversity of bacterial communities across sample compartments. Boxplots show alpha diversity by sample type based on (**A**) Chao1 richness, (**B**) Shannon diversity, and (**C**) Simpson index. *P*-values indicated results of Kruskal-Wallis tests with Bonferroni-corrected pairwise comparisons. (**D–F**) Principal coordinates analysis (PCoA) of Bray-Curtis dissimilarities reveals differences in community composition. (**D**) sample type, (**E**) site, and (**F**) plant type. PERMANOVA *P*-values and homogeneity of dispersion results are shown for each panel.

Alpha diversity showed a clear influence of site, plant compartment, and to a lesser extent, plant type (Fig. 2A through C; see Fig. S3 at https://doi.org/10.6084/m9.figshare.32002416). Chao1 richness was significantly lower at Site 1 than Sites 2 and 3 (*P* < 0.05), while Shannon and Simpson diversity did not differ significantly among sites (see Fig. S4 at https://doi.org/10.6084/m9.figshare.32002416). Diversity did not vary significantly across plant types (trees, shrubs, and small vegetation) (see Fig. S4D through F and Table S4 at https://doi.org/10.6084/m9.figshare.32002416), indicating limited influence of host growth form on richness or evenness. In contrast, sample types (see Table S5 at https://doi.org/10.6084/m9.figshare.32002416) strongly structured microbial diversity. All three indices (Chao1, Shannon, and Simpson) declined along the soil-plant continuum, with bulk soil showing the highest richness and evenness, followed by rhizosphere, roots, and shoots. Significant reductions occurred between bulk soil and both rhizosphere (Chao1: *P* = 0.039; Shannon and Simpson: *P* = 0.005) and roots (Chao1: *P* = 0.010; Shannon and Simpson: *P* < 0.001). Diversity decreased further from roots to shoots (Chao1: *P* = 0.005; Shannon and Simpson: *P* = 0.001) (Fig. 2A through C; see Table S5 at https://doi.org/10.6084/m9.figshare.32002416). Differences between rhizosphere and plant tissues were weaker and nonsignificant, indicating that the strongest filtering steps occur during transitions into the plant and from roots to aboveground tissues.

Beta-diversity analyses (Bray-Curtis) were consistent with these patterns and supported by PERMANOVA results across all factors (Fig. 2D through F; also see Table S6 at https://doi.org/10.6084/m9.figshare.32002416). Microbial communities differed significantly by sample type (*P* = 0.001; Fig. 2D) and by plant type (*P* = 0.006; Fig. 2F). In contrast, the effect by site was not statistically significant (*P* = 0.09; Fig. 2E), although it suggested a trend toward compositional differentiation. Beta-dispersion tests were also significant for each factor (*P* = 0.001), indicating that both differences in centroid positions and variability among groups contributed to the observed patterns (see Table S6 at https://doi.org/10.6084/m9.figshare.32002416). Overall, these results suggest that environmental filtering along the soil-plant continuum is the dominant force shaping microbiome differentiation, while site-level heterogeneity and host identity exert secondary influences.

### Bacterial diversity and richness respond to spatial distribution

**Fig 3:**
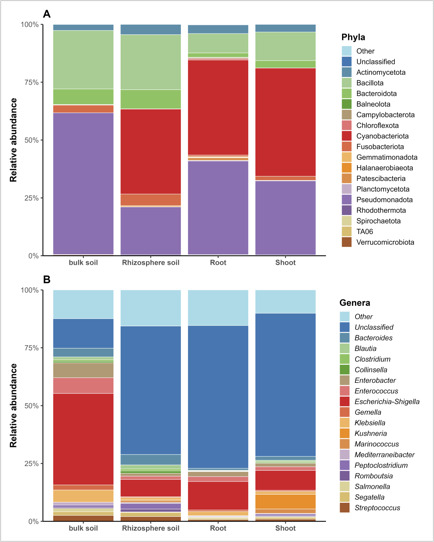
Relative abundance of dominant bacterial genera by sample type. (**A**) Mean relative abundance of dominant bacterial phyla across bulk soil, rhizosphere soil, and root and shoot samples. (**B**) Mean relative abundance of dominant genera within each compartment. Only the most abundant taxa are shown; low-abundance genera are grouped as “Other.”

Taxonomic profiling revealed clear structuring of bacterial communities across compartments (Fig. 3). At the phylum level (Fig. 3A), microbiomes were dominated by Pseudomonadota, followed by Bacillota and Cyanobacteriota with pronounced compartment-specific differences. Bulk soil was enriched in Pseudomonadota, followed by Bacteroidota. The rhizosphere soil was enriched with Cyanobacteriota, followed by Bacillota and Pseudomonadota. Root and shoot tissues showed reduced phylum-level diversity but increased dominance of Pseudomonadota and Cyanobacteriota. At the genus level (Fig. 3B), bulk soil and rhizosphere compartments displayed broad taxonomic richness, including *Escherichia Shigella, Enterococcus,* and *Enterobacter*. In contrast, rhizosphere soil and root and shoot compartments were dominated by unclassified taxa. Root and shoot were also dominated by specialized halotolerant genera, such as *Kushneria* sp., *Marinococcus* sp., and *Halomonas* sp., reflecting selective colonization of internal plant tissues. Although detailed inter-site comparisons were limited, rhizosphere communities appeared more diverse and compositionally distinct than bulk soil, with potential site-specific enrichment of certain genera (e.g., *Blautia*, *Gemella*, and *Peptoclostridium*). Further resolution of site-level variation may reveal subtle host or edaphic influences within each sabkha.

### Indicator bacterial species reveal strong compartment- and host-specific signatures

**Fig 4:**
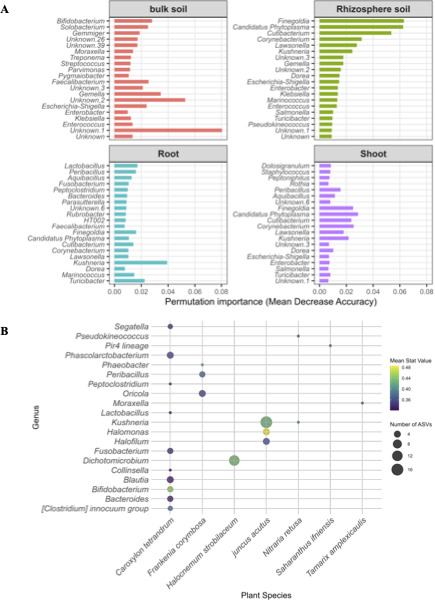
Random Forest and indicator species analyses. (**A**) Top 20 bacterial genera contributing to classification accuracy across four compartments (bulk soil, rhizosphere soil, root, and shoot), ranked by permutation importance using Random Forest. (**B**) Indicator genera identified by IndVal analysis across plant species. Dot size corresponds to the number of ASVs, and color indicates the mean IndVal score (indicator strength).

The analysis of bacterial indicator taxa across the three sabkhas revealed both distinct and overlapping microbial signatures among plant compartments and hosts (Fig. 4). Random Forest analysis identified clear compartment-specific patterns in bacterial community structure (Fig. 4A). Bulk soil communities were primarily characterized by several unclassified taxa*,* alongside genera such as *Bifidobacterium, Solobacteriu*m, and *Gemella*. The rhizosphere was enriched in *Finegoldia*, *Candidatus Phytoplasma*, *Cutibacterium*, *Corynebacterium*, and several unclassified genera. Root tissues showed strong enrichment of *Kushneria*, *Lactobacillus*, and *Turicibacter*, reflecting strong selective filtering within the endosphere. Shoot-associated communities were characterized by *Candidatus Phytoplasma*, *Finegoldia*, *Cutibacterium*, *Corynebacterium*, *Kushneria*, and *Peribacillus*. These indicator genera, which achieved high permutation importance scores, demonstrate pronounced spatial partitioning along the soil-root-shoot continuum, highlighting the distinct ecological pressures structuring each plant compartment.

To complement these patterns (Fig. 4A), we performed IndVal analysis to assess host-specific associations (Fig. 4B). *Juncus acutus* showed the strongest selective filtering, with high indicator values for the halophilic genera *Halomonas* sp. (IndVal = 0.492, *P* = 0.0004)*, Kushneria* sp. (0.452, *P* = 0.0013), and *Halofilum* sp. (0.376, *P* = 0.0099). *Halocnemum strobilaceum* exhibited a single but highly robust indicator genus*, Dichotomicrobium* (IndVal = 0.448, *P* = 0.0018), consistent with its adaptation to extreme salinity and nutrient-poor soils. *Frankenia corymbosa* plant was enriched in *Phaeobacter* sp. (IndVal = 0.401, *P* = 0.0056), *Peribacillus* sp. (0.396, *P* = 0.0077), and *Oricola* sp. (0.357, *P* = 0.0245), and *Nitraria retusa* hosted two distinct indicators, *Kushneria* sp. (IndVal = 0.387, *P* = 0.0125) and *Pseudokineococcuss sp*. (0.362, *P* = 0.0148), whereas *Saharanthus ifniensis* and *Tamarix amplexicaulis* displayed a single association, for instance, Pir4 lineage (IndVal = 0.374, *P* = 0.0107) and *Moraxella* sp. (IndVal = 0.363, *P* = 0.0134) respectively. *Caroxylon tetrandrum* hosted the most diverse (10 genera) indicator profile, including *Bifidobacterium* sp. (IndVal = 0.469, *P* = 0.0002), *Clostridium innocuum* group (0.379, *P* = 0.0071), *Phascolarctobacterium* sp. (0.377, *P* = 0.0047), *Fusobacterium* sp. (0.356, *P* = 0.0113), *Segatella* sp. (0.355, *P* = 0.0126), *Bacteroides* sp*., Peptoclostridium* sp*., Blautia* sp*., Collinsella* sp*.,* and *Lactobacillus* sp. (Fig. 4B). Together, these results indicate that while environmental filtering dominates microbiome assembly, host identity modulates the specificity and breadth of microbial recruitment under extreme sabkha conditions.

### Core bacteriome and cross-host convergence

To identify conserved bacterial taxa across plant functional types (shrubs, small vegetation, and trees), we profiled the core genera within the shoot, root, and rhizosphere compartments (see Fig. S5A through C at https://doi.org/10.6084/m9.figshare.32002416) and assessed ASV-level diversity to evaluate cross-compartment microbial convergence (see Fig. S5D at https://doi.org/10.6084/m9.figshare.32002416). Because this analysis integrated all ASVs across three sites and plant hosts, the detected core taxa represent across site and across host plants patterns rather than site-specific subsets. In the shoot compartment (see Fig. S5A at https://doi.org/10.6084/m9.figshare.32002416), *Kushneria* sp.*, Enterobacter* sp.*, Escherichia-Shigella* sp., and *Marinococcus* sp. were consistently detected across all plant types, with small vegetation exhibiting the highest core genus richness and relative abundance. Shrub shoots displayed slightly lower diversity, suggesting stronger selective pressures in aboveground tissues of woody perennials. Root-associated bacterial communities (see Fig. S5B at https://doi.org/10.6084/m9.figshare.32002416) showed a comparable core structure with dominated *Fusobacterium* sp.*, Enterococcus* sp.*,* and *Peptoclostridium* sp. Tree roots harbored the richest and most evenly distributed core bacteriome, potentially reflecting more stable or complex rhizosphere-root interactions associated with perennial root systems. In rhizosphere soil (see Fig. S5C at https://doi.org/10.6084/m9.figshare.32002416), broader taxonomic diversity was observed, with *Escherichia-Shigella, Enterobacter* sp., and *Gemella* sp. dominating across all plant types. Trees again exhibited higher genus richness and compositional complexity than shrubs and small vegetation, consistent with increasing structured rhizosphere environments beneath long-lived plant canopies.

ASV-level intersections revealed that 66% of ASVs were shared among roots, shoots, and rhizosphere soils (see Fig. S5D at https://doi.org/10.6084/m9.figshare.32002416), indicating a robust cross-site microbial backbone. Root-specific ASVs accounted for 14%, whereas shoot-specific ASVs represented only 1%. No unique ASVs were detected in the rhizosphere after applying identical normalization and filtering criteria across all compartments, which indicated that this pattern was not driven by analytical thresholds. Instead, the high degree of ASV sharing suggests strong overlap between bulk soil and rhizosphere communities in these extreme saline environments where deterministic environmental filtering may constrain niche differentiation and promote microbial recruitment from a shared halotolerant pool. Together, these findings distinguish stable, environmentally selected core taxa from more context-dependent, host- or microhabitat-associated members.

### Environmental drivers of community structure

**Fig 5:**
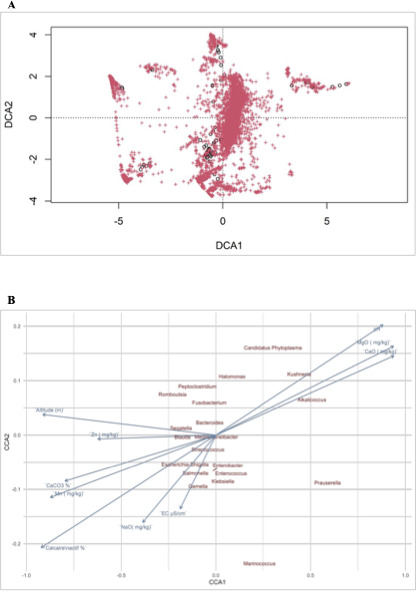
Ordination analyses of bacterial community structure and its relationship with environmental variables. (**A**) Detrended correspondence analysis (DCA) showing overall patterns of variation in bacterial community composition across samples. (**B**) Canonical correspondence analysis (CCA) of bacterial community structure constrained by environmental variables. Arrows represent soil physicochemical parameters, while red labels indicate bacterial genera.The direction and length of the arrows indicate the strength and direction of each environmental gradient influencing community composition, with factors such as such as EC, Na_2_O, MgO, and CaO showed strong correlations with distribution of specific bacterial taxa.

To elucidate the environmental factors shaping bacterial community composition in the sabkhas, we first assessed the underlying species response model using detrended correspondence analysis (DCA). The length of the first DCA axis was high (10.89 SD units), indicating strong species turnover and unimodal responses to environmental gradients (Fig. 5A; see Table S7 at https://doi.org/10.6084/m9.figshare.32002416) and thereby justifying the use of canonical correspondence analysis (CCA). The broad dispersion of taxa along DCA1 further suggests pronounced ecological differentiation among bacterial assemblages across sabkha environments, consistent with strong environmental filtering. Subsequently, CCA revealed clear relationships between dominant bacterial genera and soil physicochemical properties across sabkha environments (Fig. 5B; see Table S8 at https://doi.org/10.6084/m9.figshare.32002416). Salinity, alkalinity, and calcareousness emerged as the strongest environmental correlates influencing bacterial community composition across sabkha soils (see Table S8 at https://doi.org/10.6084/m9.figshare.32002416). Electrical conductivity (EC), soil pH, and both total and active carbonate fractions were significantly associated with community structure (*P* = 0.001). Genera such as *Kushneria, Halomonas*, and *Marinococcus* were strongly aligned with high EC and carbonate gradients, reflecting their prevalence in saline-alkaline soils. In contrast, *Escherichia-Shigella and Enterococcus* were associated with nutrient-rich contexts under high EC conditions. Together, these results confirm that abiotic filters imposed by soil physicochemistry are the dominant drivers of sabkha bacteriome assembly.

### Culture-dependent isolation and identification of halophytic bacteria

**Fig 6:**
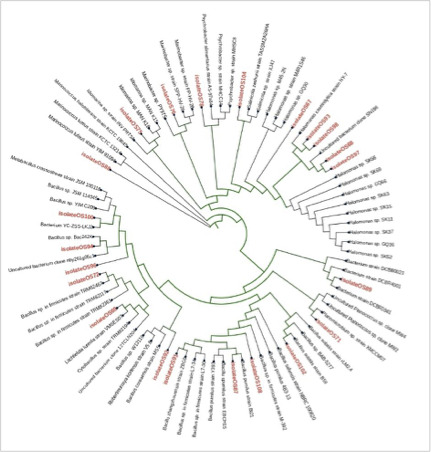
Phylogenetic placement of sabkha bacterial isolates. Maximum-likelihood phylogenetic tree based on 16S rRNA gene sequences showing the relationships between sabkha-derived bacterial isolates (highlighted in red) and reference strains retrieved from the NCBI database. Isolates clustered within well-known halotolerant and halophilic genera, including *Halomonas* spp., *Bacillus* spp., *Salinicola* spp., *Marinobacter* spp., *Psychrobacter* spp., and *Marinococcus* spp. Bootstrap support values greater than 70% are shown at the corresponding nodes.

To complement the culture-independent profiling of bacterial communities and validate the presence of dominant bacterial taxa in the sabkha ecosystem, we implemented a culture-dependent approach aimed at isolating halotolerant bacteria from key halophytes plants, *Juncus acutus* and *Halocnemum strobilaceum*, which were selected based on their distinctive signatures of indicator genera (Fig. 4) and core taxa (see Fig. S5 at https://doi.org/10.6084/m9.figshare.32002416). Root, shoot, and rhizosphere samples yielded 19 morphologically distinct isolates. Sanger sequencing followed by phylogenetic analysis (Fig. 6) assigned these isolates to several known halophilic or halotolerant taxa, including *Halomonas*, *Bacillus*, *Salinicola*, *Marinobacter*, *Psychrobacter,* and *Marinococcus*. The recovered isolates represent a diverse set of halophilic and halotolerant bacteria associated with sabkha halophytes (Table 1). *Halomonas* isolates (GQ30 and SK63) from *Halocnemum strobilaceum* showed sequence identity (96.4%–98.2%) to described halophilic species and demonstrated robust growth on Marine Agar, indicating strong salt tolerance. Additional halophilic taxa included *Idiomarina* sp. MAN K15 and *Marinobacter* sp. PY97S, both isolated from *Halocnemum* shoots. Other isolates, such as *Psychrobacter alimentarius* Psys_23 from *Juncus* roots, a psychrophilic yet halotolerant species, and *Bacillus pumilus* AM2 from *Halocnemum* shoots, are known for broad stress tolerance. Further isolates included *Planomicrobium* sp. XMCCM07 and *Bacillus* sp. TRM82117 from *Juncus* roots, along with an uncultured bacterium and an unidentified strain (DCBB0021) from *Halocnemum*, expanding the phylogenetic breadth of culturable sabkha-associated bacteria.

**TABLE 1:** Summary of cultured halophilic and halotolerant bacterial isolates recovered from sabkha halophytes^*a*^

Identified isolates	% identical sites	% coverage	Source	Salt tolerance	Key reference
*Halomonas* sp. GQ30	96.4	100%	*Halocnemum* rhizosphere	Halophilic (5%–25% NaCl)	(39)
*Halomonas* sp. SK63	98.2	100%	*Halocnemum* root	Halophilic	(40)
Uncultured bacterium	91.9	99.18%	–^*b*^	–	–
*Planomicrobium* sp. XMCCM07	99.1	100%	*Juncus* root	–	–
*Bacillus* sp. TRM82117	88.4	100%	*Juncus* root	–	–
*Idiomarina* sp. MAN K15	97.9%	99.83%	*Halocnemum* shoot	Moderate halophile	(41)
*Marinobacter* sp. PY97S	97.1%	100%	*Halocnemum* shoot	Halophilic	–
*Psychrobacter alimentarius* Psys_23	97.7%	99.76%	*Juncus* root	Psychrophilic and halotolerant	–
*Bacillus pumilus* AM2	99.7%	100%	*Halocnemum* shoot	Halotolerant	(42)
Bacterium strain DCBB0021	99.2%	100%	*Halocnemum* root	–	–
*Bacillus* sp. L7-30	99.1%	100%	*Halocnemum* root	Halotolerant	–
*Bacillus* sp. Y1	98.4%	100%	*Halocnemum* rhizosphere	Halotolerant	–
*Halomonas* sp. SK52	97.7%	100%	*Halocnemum* root	Halophilic	–
Bacterium YC-ZSS-LKJ2 (isolate 1)	99.0%	100%	*Halocnemum* rhizosphere	Halotolerant	–
*Halomonas* sp. GQ66	97.7%	100%	*Halocnemum* root	Halophilic	–
*Halomonas* sp. SK15	98.0%	100%	*Halocnemum* root	Halophilic	–
*Bacillus* sp. BAB-5277	98.2%	100%	Juncus shoot	Halotolerant	–
*Salinicola* sp. XJ47	98.8%	97.01%	Juncus shoot (1)	Halophilic	Salinicola traits
*Bacillus safensis* F6	99.4%	100%	Juncus root (1)	Halotolerant	Bacillus traits

^*a*^ Table includes taxonomic identifications, sequence identity and coverage, source compartment, salt tolerance traits (halophilic/halotolerant), and relevant references. Isolates were identified based on 16S rRNA gene sequence similarity to reference strains in NCBI.

^*b*^ –, not applicable.

Phylogenetic analysis revealed that sabkha isolates cluster within major halophilic and stress-adapted lineages (Fig. 6). Many isolates grouped with *Halomonas*, *Idiomarina*, and *Marinobacter*, while others aligned with halotolerant Firmicutes such as *Bacillus* and *Planomicrobium*. Additional isolates branched with *Psychrobacter* and several uncultured taxa, highlighting both known and previously uncharacterized diversity within sabkha microbiomes. Notably, many of these genera overlapped with dominant and indicator taxa identified through sequencing, providing independent confirmation of their prevalence in sabkha environments and demonstrating strong concordance between culture-independent and culture-based approaches. The phylogeny (Fig. 6) underscores the broad taxonomic range and strong enrichment of stress-adapted bacteria associated with halophytes in extreme desert saline ecosystems.

## DISCUSSION

We found that sabkha ecosystems exert strong environmental filtering on bacterial communities, producing highly structured microbiomes across compartments and plant types. Across all sabkhas, pronounced gradients in ionic composition (Na₂O and K₂O), salinity, and pH corresponded to marked shifts in bacterial diversity and composition. We observed a consistent transition from taxonomically rich bulk soil microbiomes toward increasingly selective assemblages within the rhizosphere, root, and shoot, demonstrating that sabkha habitats impose stringent constraints on microbial colonization. The occurrence of unclassified taxa in this study likely reflects the ecological novelty and limited prior microbiome characterization of southern Moroccan sabkha ecosystems. These extreme and underexplored habitats are expected to harbor previously undescribed or poorly represented microbial lineages that are not yet comprehensively captured in current reference databases. Thus, the presence of unclassified ASVs should be interpreted as indicative of database limitations and the uniqueness of these environments rather than as a methodological artifact of sequencing length alone.

### Environmental filtering dominated bacterial communities

Our findings demonstrate that microbial community assembly in sabkha habitats is overwhelmingly shaped by abiotic forces, supporting our first hypothesis. Extreme salinity, high carbonate content, and a consistently alkaline pH characterized the bulk soils across all three sabkha sites. These conditions impose substantial selective pressure on microbial physiology (43). Alpha diversity declined progressively from bulk soil to rhizosphere, root, and shoot compartments, revealing hierarchical filtering along the soil-plant continuum. Beta diversity analyses further supported this trend, showing strong compartmental separation driven by differences in both community centroids and dispersion. Although hierarchical filtering has been widely reported across terrestrial ecosystems (7, 44), the intensity of filtering observed in sabkhas was particularly pronounced, reflecting the extreme environmental constraints shaping microbial community assembly in these saline desert ecosystems.

### Shared halotolerant core microbiota linking hosts and compartments

Despite edaphic heterogeneity among sites, bacterial communities converged on a stable halotolerant core composed primarily of *Halomonas*, *Kushneria*, *Marinococcus,* and related genera that are well known for osmoprotection, salt tolerance, and metabolic plasticity (45, 46). This strong cross-site support provides clear evidence for the second hypothesis, indicating deterministic environmental filtering as the major factor shaping microbiome convergence. Remarkably, 66% of ASVs were shared across rhizosphere, root, and shoot compartments, underscoring the persistence of a cross-compartment microbial foundation. No unique rhizosphere ASVs were detected following uniform normalization and filtering procedures, suggesting substantial overlap between bulk soil and rhizosphere communities in these extreme saline environments. Rather than indicating ecological absence, this pattern likely reflects strong environmental restraints that limit niche diversification and promote recruitment of a shared halotolerant pool (47). In contrast, the presence of unique ASVs in root compartments reflects the strongest selective bottleneck along the continuum (48). Perennial shrubs consistently hosted the richest and most phylogenetically diverse core microbiota across compartments, likely due to their long lifespan, deeper rooting systems, and more stable rhizosphere environments (20, 49).

### Host effects exist but remain secondary to strong environmental filtering

Although plant identity contributed to the structure of sabkha bacterial communities, its influence was modest compared to the dominant effect of edaphic filtering, in agreement with the first hypothesis. Across all sites, salinity, ionic composition, and alkalinity exerted the strongest constraints, overshadowing host-driven selection, a pattern widely observed in arid and saline environments (50–53). Plant species composition differed across the three sites (see Table S1 at https://doi.org/10.6084/m9.figshare.32002416), yet a substantial proportion of core taxa (66%) were shared across compartments and hosts (Fig. 5D), indicating cross-site and cross-host robustness. Genera such as *Halomonas, Kushneria,* and *Marinococcus* were consistently detected across sites and plant types, supporting the interpretation that strong deterministic environmental filtering under saline-alkaline conditions drives microbiome convergence in sabkha. Host genotype did produce detectable but subtle signals: for example, *Dichotomicrobium* was enriched in the roots of *Halocnemum strobilaceum*, while indicator taxa such as *Kushneria* and *Halomonas* were associated with *Juncus acutus* and *Peptoclostridium* with *Frankenia thymifolia*. Similar compartment-specific host effects, more pronounced in the endosphere than in bulk soil or rhizosphere, have been reported in multiple systems, where root exudate chemistry, tissue structure, or plant immune responses shape a subset of microbial taxa (54–57). Notably, taxa unique to root compartments (14%) represent more context-dependent members likely influenced by host-mediated filtering or microhabitat specialization.

However, these host-associated signatures were exceptions rather than the prevailing pattern. The rhizosphere and root microbiota in sabkhas were overwhelmingly structured by abiotic gradients, consistent with global trends in which soil salinity, pH, carbonate content, and aridity explain most variation in microbial community composition, whereas plant identity explains only a minor fraction (58, 59). Moreover, under extreme environmental stress, such as high ionic strength, osmotic pressure, and desiccation, host-driven selection becomes secondary to strong environmental constraints, leading to convergence toward a stable halotolerant core (50, 60). Taken together, our results indicated that while host traits may modulate the abundance of bacterial lineages, salinity and related edaphic factors remain the dominant forces shaping microbiome assembly across sabkha plant species.

### Environmental determinants of bacterial community structure

CCA results further confirmed the central role of edaphic filtering. Salinity-related parameters, particularly EC, Na₂O, K₂O, and carbonate fractions, were the strongest predictors of bacterial community composition. These gradients aligned with halophilic genera such as *Kushneria*, *Halomonas*, and *Marinococcus*, taxa characterized by osmo-adaptive mechanisms including compatible solute biosynthesis (ectoine and hydroxyectoine), active transport systems, and ion homeostasis (61, 62). Their consistent detection across sites and plants reinforces the interpretation of adaptive convergence driven by deterministic environmental filtering. In contrast, copiotrophic taxa such as *Escherichia-Shigella* and *Enterococcus* occurred in nutrient-enriched microhabitats within high EC. It is noteworthy that several genera commonly associated with animal or human microbiota (e.g., *Escherichia-Shigella, Enterococcus,* and *Corynebacterium*) were detected in specific microhabitats. Although such taxa can be flagged as contaminants in low-biomass studies, their presence in sabkha ecosystems is ecologically plausible. The study sites are actively grazed by camels, goats, and cattle, and we directly observed active grazing as well as bones and dung deposits during field sampling. These pastoral activities can introduce gut-associated microorganisms into soils and plant compartments, where they may persist under suitable conditions (63–65). Recent work also emphasizes the importance of interpreting host-associated taxa within their environmental context (65). These patterns parallel findings from other hypersaline systems (66), underscoring the global consistency of abiotic filtering under extreme salinity. Overall, these results revealed a clear ecological hierarchy: stable halotolerant core taxa reflect strong environmental filtering and cross-site convergence, whereas more variable taxa are shaped by localized nutrient inputs, microhabitat heterogeneity, and host-associated processes.

### Culture-dependent isolation confirmed stress-adapted bacterial taxa

Culture-dependent analyses provided strong support for the third hypothesis, demonstrating that dominant halophilic taxa detected via metabarcoding are also recoverable through cultivation. Nineteen morphologically distinct isolates were obtained from *J. acutus* and *H. strobilaceum* representing well-known halophilic and halotolerant genera (*Halomonas*, *Idiomarina*, *Marinobacter*, *Psychrobacter*, *Planomicrobium,* and *Bacillus*). These isolates exhibited robust growth on saline Marine Agar media, confirming their salt tolerance. Notably, several closely related species within these genera have been reported in the literature to tolerate salinity levels up to 25% NaCl, consistent with established halophilic physiology (40, 41, 67). Stress-tolerant Bacillota such as *Bacillus pumilus* and *Planomicrobium* were also recovered, consistent with their resilience in desert environments (68). The correspondence between cultured-independent and culture-based results demonstrates both the ecological dominance and culturable robustness of sabkha halophiles. The isolation of strains clustering with uncultured lineages additionally highlights sabkhas as reservoirs of yet unexplored microbial diversity with promising biotechnological potential.

### Alignment with global patterns of the halophyte-associated bacteriome

Our findings mirror global patterns observed in halophytic systems. A recent meta-analysis (69) identified *Thalassospira*, *Erythrobacter,* and *Marinobacter* as predictive halophilic taxa in saline soils, echoing the sabkha core assemblage. Similarly, studies from Utah desert halophytes consistently isolated *Halomonas*, *Kushneria*, and *Bacillus* (70), many of which exhibit extreme salt tolerance (up to 4 M NaCl) and promote plant performance under salinity stress. The strong overlap between our sabkha core microbiome and halophilic lineages reported globally underscores deterministic environmental filtering as a pervasive force shaping plant-microbe associations across saline habitats worldwide.

### Conclusion

This study provides a comprehensive characterization of bacterial communities associated with native halophytes in sabkha ecosystems of southern Morocco. Microbiome assembly in these hypersaline, alkaline habitats is shaped primarily by deterministic environmental filtering, with deep salinity, ionic, and carbonate gradients driving a progressive transition from diverse bulk soil microbiota to increasingly selective rhizosphere, root, and shoot communities. Despite site-level heterogeneity, all plant hosts shared a conserved halotolerant core dominated by *Halomonas*, *Kushneria*, and *Marinococcus*, while host identity exerted only a secondary influence. The substantial overlap of taxa across compartments highlights the root interior as the most selective microhabitat. Culture-dependent isolation further validated these patterns and recovered culturable representatives of dominant halophilic lineages.

Ecologically, sabkhas emerge as valuable natural laboratories for studying microbial adaptation to extreme abiotic stress. The communities identified here are enriched for traits associated with osmoadaptation, desiccation tolerance, and general stress resilience, providing insights into the mechanisms underpinning survival in hypersaline desert environments. However, the taxonomic and functional resolution of 16S rRNA sequencing limits our ability to identify strain-level adaptive features and specific metabolic pathways. Future metagenomic, metatranscriptomic, and physiological studies will be essential to resolve the genetic basis of halotolerance, especially genes involved in compatible-solute biosynthesis, membrane stabilization, and ion homeostasis. Moreover, although core and indicator taxa were identified, their functional roles remain inferential. Experimental validation through plant inoculation assays and *in situ* manipulations will be necessary to determine whether these halophilic lineages enhance plant performance under salinity and alkalinity stress. Altogether, the resilient halotolerant taxa highlighted in this study represent promising candidates for the development of targeted microbial inoculants and synthetic consortia (SynComs) aimed at improving crop productivity in saline and alkaline soils. Integrating environmental microbiology with functional genomics and ecological experimentation will pave the way for sustainable biotechnological applications in arid and salt-affected agricultural systems.

## Data Availability

The amplicon data sets used in this study are available in the NCBI Sequence Read Archive database under accession number PRJNA1395314.

## References

[1] Guevara-Luna J, Arroyo-Herrera I, Tapia-García EY, Estrada-de Los Santos P, Ortega-Nava AJ, Vásquez-Murrieta MS. 2024. Diversity and biotechnological potential of cultivable halophilic and halotolerant bacteria from the “Los Negritos” geothermal area. Microorganisms 12:482. doi:10.3390/microorganisms12030482 38543532 PMC10972316

[2] Debbagh-Nour H, Khourchi S, Mouttaqi AE, Elfermi R, Bourazza A, Malou OP, Ducousso M, Boukcim H, Hijri M, Hirich A. 2025. Plant identity and environmental filtering are the key drivers of bacterial community structure in four desert plant species from the Sahara Desert in Morocco. BMC Microbiol 25:734. doi:10.1186/s12866-025-04463-w 41225325 PMC12613618

[3] Blakney AJC, Bainard LD, St-Arnaud M, Hijri M. 2023. Soil chemistry and soil history significantly structure oomycete communities in Brassicaceae crop rotations. Appl Environ Microbiol 89:e0131422. doi:10.1128/aem.01314-22 36629416 PMC9888183

[4] Liu Y, Xun W, Chen L, Xu Z, Zhang N, Feng H, Zhang Q, Zhang R. 2022. Rhizosphere microbes enhance plant salt tolerance: toward crop production in saline soil. Comput Struct Biotechnol J 20:6543–6551. doi:10.1016/j.csbj.2022.11.046 36467579 PMC9712829

[5] Song S, Yang X, Zhu J, Tang R, Tang Z. 2025. Plant functional diversity regulates the composition and diversity of soil microbial communities in temperate grasslands of northern China. Funct Ecol 39:2314–2326. doi:10.1111/1365-2435.70103

[6] Trivedi P, Leach JE, Tringe SG, Sa T, Singh BK. 2020. Plant-microbiome interactions: from community assembly to plant health. Nat Rev Microbiol 18:607–621. doi:10.1038/s41579-020-0412-1 32788714

[7] Shao L, Li X, Xiao T, Lu T, Li J, Deng J, Xiao E. 2023. Variations in microbial assemblage between rhizosphere and root endosphere microbiomes contribute to host plant growth under cadmium stress. Appl Environ Microbiol 89:e0096023. doi:10.1128/aem.00960-23 37855640 PMC10686079

[8] Sauma-Sánchez T, Alcorta J, Tamayo-Leiva J, Díez B, Bezuidenhout H, Cowan DA, Ramond J-B. 2024. Functional redundancy buffers the effect of poly-extreme environmental conditions on southern African dryland soil microbial communities. FEMS Microbiol Ecol 100:fiae157. doi:10.1093/femsec/fiae157 39568064 PMC11636270

[9] Subedi SC, Epps S, Ankrah N, Bhandari S. 2025. Soil microbes’ role in plant germination and growth under salt stress. J Environ Manage 386:125841. doi:10.1016/j.jenvman.2025.125841 40382931

[10] Tiwari S, Sharma B, Bisht N, Tewari L. 2023. Role of beneficial microbial gene pool in mitigating salt/nutrient stress of plants in saline soils through underground phytostimulating signalling molecules. Pedosphere 33:153–171. doi:10.1016/j.pedsph.2022.06.029

[11] Aqeel M, Ran J, Hu W, Irshad MK, Dong L, Akram MA, Eldesoky GE, Aljuwayid AM, Chuah LF, Deng J. 2023. Plant-soil-microbe interactions in maintaining ecosystem stability and coordinated turnover under changing environmental conditions. Chemosphere 318:137924. doi:10.1016/j.chemosphere.2023.137924 36682633

[12] Chai Y, Cao Y, Yue M, Tian T, Yin Q, Dang H, Quan J, Zhang R, Wang M. 2019. Soil abiotic properties and plant functional traits mediate associations between soil microbial and plant communities during a secondary forest succession on the Loess Plateau. Front Microbiol 10:895. doi:10.3389/fmicb.2019.00895 31105679 PMC6499021

[13] Fahey C, Koyama A, Antunes PM, Dunfield K, Flory SL. 2020. Plant communities mediate the interactive effects of invasion and drought on soil microbial communities. ISME J 14:1396–1409. doi:10.1038/s41396-020-0614-6 32076127 PMC7242364

[14] Singh M, Singh SK, Sharma JG, Giri B. 2024. Insights into the multifaceted roles of soil microbes in mitigating abiotic stress in crop plants: a review. Environ Exp Bot 228:106010. doi:10.1016/j.envexpbot.2024.106010

[15] Arshad MJ, Khan MI, Ali MH, Farooq Q, Hussain MI, Seleiman MF, Asghar MA. 2024. Enhanced wheat productivity in saline soil through the combined application of poultry manure and beneficial microbes. BMC Plant Biol 24:423. doi:10.1186/s12870-024-05137-x 38760709 PMC11102207

[16] Liu F, Hewezi T, Lebeis SL, Pantalone V, Grewal PS, Staton ME. 2019. Soil indigenous microbiome and plant genotypes cooperatively modify soybean rhizosphere microbiome assembly. BMC Microbiol 19:201. doi:10.1186/s12866-019-1572-x 31477026 PMC6720100

[17] Zeng Q, Hu H-W, Ge A-H, Xiong C, Zhai C-C, Duan G-L, Han L-L, Huang S-Y, Zhang L-M. 2025. Plant-microbiome interactions and their impacts on plant adaptation to climate change. J Integr Plant Biol 67:826–844. doi:10.1111/jipb.13863 39981843

[18] Fierer N. 2017. Embracing the unknown: disentangling the complexities of the soil microbiome. Nat Rev Microbiol 15:579–590. doi:10.1038/nrmicro.2017.87 28824177

[19] Lebeis SL, Paredes SH, Lundberg DS, Breakfield N, Gehring J, McDonald M, Malfatti S, Glavina del Rio T, Jones CD, Tringe SG, Dangl JL. 2015. Salicylic acid modulates colonization of the root microbiome by specific bacterial taxa. Science 349:860–864. doi:10.1126/science.aaa8764 26184915

[20] Bertola M, Righetti L, Gazza L, Ferrarini A, Fornasier F, Cirlini M, Lolli V, Galaverna G, Visioli G. 2023. Perenniality, more than genotypes, shapes biological and chemical rhizosphere composition of perennial wheat lines. Front Plant Sci 14:1172857. doi:10.3389/fpls.2023.1172857 37223792 PMC10200949

[21] Maestre FT, Quero JL, Gotelli NJ, Escudero A, Ochoa V, Delgado-Baquerizo M, García-Gómez M, Bowker MA, Soliveres S, Escolar C, et al.. 2012. Plant species richness and ecosystem multifunctionality in global drylands. Science 335:214–218. doi:10.1126/science.1215442 22246775 PMC3558739

[22] Whitaker F, Mey Didi-Ooi S, Jameson J, Strohmenger CJ. 2014. Origins of evaporites in a holocene mixed clastic and carbonate coastal sabkha: preliminary hydrological and geochemical data from Mesaieed Sabkha, Qatar. IPTC 2014; Doha, Qatar European Association of Geoscientists & Engineers

[23] Wickham H. 2016. Programming with ggplot2, p 241–253. *In* ggplot2: elegant graphics for data analysis. Springer.

[24] Pebesma E. 2018. Simple features for R: standardized support for spatial vector data. R J 10:439. doi:10.32614/RJ-2018-009

[25] Massicotte P, South A, Hufkens K. 2023. Package rnaturalearth: world map data from natural earth

[26] Singh R, Pandey KD, Singh M, Singh SK, Hashem A, Al-Arjani A-BF, Abd Allah EF, Singh PK, Kumar A. 2022. Isolation and characterization of endophytes bacterial strains of Momordica charantia L. and their possible approach in stress management. Microorganisms 10:290. doi:10.3390/microorganisms10020290 35208743 PMC8877101

[27] Koczorski P, Furtado BU, Gołębiewski M, Hulisz P, Thiem D, Baum C, Weih M, Hrynkiewicz K. 2022. Mixed growth of Salix species can promote phosphate-solubilizing bacteria in the roots and rhizosphere. Front Microbiol 13:1006722. doi:10.3389/fmicb.2022.1006722 36338053 PMC9634750

[28] Ahmed B, Nazari M, Legeay J, Schwinghamer T, Hijri M, Smith D. 2025. The path effects of a bacterial signal compound on the microbiome of canola. BMC Plant Biol 25:976. doi:10.1186/s12870-025-06929-5 40730957 PMC12305995

[29] Callahan BJ, McMurdie PJ, Rosen MJ, Han AW, Johnson AJA, Holmes SP. 2016. DADA2: high-resolution sample inference from Illumina amplicon data. Nat Methods 13:581–583. doi:10.1038/nmeth.3869 27214047 PMC4927377

[30] Straub D, Blackwell N, Langarica-Fuentes A, Peltzer A, Nahnsen S, Kleindienst S. 2020. Interpretations of environmental microbial community studies are biased by the selected 16S rRNA (gene) amplicon sequencing pipeline. Front Microbiol 11:550420. doi:10.3389/fmicb.2020.550420 33193131 PMC7645116

[31] Quast C, Pruesse E, Yilmaz P, Gerken J, Schweer T, Yarza P, Peplies J, Glöckner FO. 2013. The SILVA ribosomal RNA gene database project: improved data processing and web-based tools. Nucleic Acids Res 41:D590–6. doi:10.1093/nar/gks1219 23193283 PMC3531112

[32] Oksanen J, Simpson GL, Blanchet FG, Kindt R, Legendre P, Minchin PR, O’Hara RB, Solymos P, Stevens MHH, Szoecs E, et al.. 2013. Package ‘vegan’. Community ecology package 2:1–295.

[33] McMurdie PJ, Holmes S. 2013. phyloseq: an R package for reproducible interactive analysis and graphics of microbiome census data. PLoS One 8:e61217. doi:10.1371/journal.pone.0061217 23630581 PMC3632530

[34] Kassambara A. 2016. Ggpubr:“ggplot2” based publication ready plots (p. 0.6. 0)[Dataset]. CRAN package ggpubr

[35] Liaw A, Wiener M. 2002. Classification and regression by randomForest. R news 2:18–22.

[36] Cáceres MD, Legendre P. 2009. Associations between species and groups of sites: indices and statistical inference. Ecology 90:3566–3574. doi:10.1890/08-1823.1 20120823

[37] Hill MO, Gauch HG Jr. 1980. Detrended correspondence analysis: an improved ordination technique. Vegetatio 42:47–58. doi:10.1007/BF00048870

[38] Legendre P, Legendre L. 2012. Numerical ecology. Vol. 24. Elsevier.

[39] Mousa AAA, Mahmoud WH, Elsaied HE, Elbeltagy AE. 2025. Halomonas sp for sustainable agriculture a potential halo bio fertilizer for tomato plants with biocontrol activity against Fusarium wilt under saline environments. Sci Rep 15:30748. doi:10.1038/s41598-025-12974-9 40841562 PMC12370916

[40] Ventosa A, Nieto JJ, Oren A. 1998. Biology of moderately halophilic aerobic bacteria. Microbiol Mol Biol Rev 62:504–544. doi:10.1128/MMBR.62.2.504-544.1998 9618450 PMC98923

[41] Ivanova EP, Romanenko LA, Chun J, Matte MH, Matte GR, Mikhailov VV, Svetashev VI, Huq A, Maugel T, Colwell RR. 2000. Idiomarina gen. nov., comprising novel indigenous deep-sea bacteria from the Pacific Ocean, including descriptions of two species, Idiomarina abyssalis sp. nov. and Idiomarina zobellii sp. nov. Int J Syst Evol Microbiol 50 Pt 2:901–907. doi:10.1099/00207713-50-2-901 10758902

[42] Dobrzyński J, Jakubowska Z, Dybek B. 2022. Potential of Bacillus pumilus to directly promote plant growth. Front Microbiol 13:1069053. doi:10.3389/fmicb.2022.1069053 36620067 PMC9810630

[43] Kumawat C, Kumar A, Parshad J, Sharma SS, Patra A, Dogra P, Yadav GK, Dadhich SK, Verma R, Kumawat GL. 2022. Microbial diversity and adaptation under salt-affected soils: a review. Sustainability 14:9280. doi:10.3390/su14159280

[44] Edwards J, Johnson C, Santos-Medellín C, Lurie E, Podishetty NK, Bhatnagar S, Eisen JA, Sundaresan V. 2015. Structure, variation, and assembly of the root-associated microbiomes of rice. Proc Natl Acad Sci USA 112:E911–E920. doi:10.1073/pnas.1414592112 25605935 PMC4345613

[45] Hamdene I, Bez C, Bertani I, López-Menchero JR, Yahyaoui A, Venturi V, Sadfi-Zouaoui N. 2025. Endophytic bacterial communities associated with halophytic plants in kebili and Gabes regions of Southern Tunisia. BMC Microbiol 25:683. doi:10.1186/s12866-025-04309-5 41131465 PMC12548130

[46] Mapelli F, Marasco R, Rolli E, Barbato M, Cherif H, Guesmi A, Ouzari I, Daffonchio D, Borin S. 2013. Potential for plant growth promotion of rhizobacteria associated with Salicornia growing in Tunisian hypersaline soils. Biomed Res Int 2013:248078. doi:10.1155/2013/248078 23781499 PMC3679824

[47] Wang P, Yang L, Sun J, Yang Y, Qu Y, Wang C, Liu D, Huang L, Cui X, Liu Y. 2022. Structure and function of rhizosphere soil and root endophytic microbial communities associated with root rot of Panax notoginseng. Front Plant Sci 12:752683. doi:10.3389/fpls.2021.752683 35069616 PMC8766989

[48] Cao H, Xu L, Song J, Xun M, Zhang W, Yang H. 2024. Bacterial community structure and co-occurrence networks in the rhizosphere and root endosphere of the grafted apple. BMC Microbiol 24:53. doi:10.1186/s12866-024-03210-x 38341527 PMC10858598

[49] Michl K, Kanasugi M, Förster A, Wuggenig R, Issifu S, Hrynkiewicz K, Emmerling C, David C, Dumont B, Mårtensson L-MD, Rasche F, Berg G, Cernava T. 2025. The microbiome of a perennial cereal differs from annual winter wheat only in the root endosphere. ISME Commun 5:ycae165. doi:10.1093/ismeco/ycae165 39936170 PMC11812607

[50] Addison SL, Yan Z-Z, Carlin T, Rúa MA, Smaill SJ, Daley K, Singh BK, Wakelin SA. 2025. Unravelling changes in the Pinus radiata root and soil microbiomes as a function of aridity. Glob Chang Biol 31:e70165. doi:10.1111/gcb.70165 40200676 PMC11979571

[51] Hodgson RJ, Liddicoat C, Cando-Dumancela C, Fickling NW, Peddle SD, Ramesh S, Breed MF. 2024. Increasing aridity strengthens the core bacterial rhizosphere associations in the pan-palaeotropical C4 grass, Themeda triandra. Appl Soil Ecol 201:105514. doi:10.1016/j.apsoil.2024.105514

[52] Rahman S, Ahmad M, Aziz MA, Alrayssi TI, Mohammad AS, Alothman R, Masmoudi K. 2025. Exploring the bacterial communities in date palm roots in saline versus non-saline environment. BMC Plant Biol 25:855. doi:10.1186/s12870-025-06882-3 40610889 PMC12224762

[53] Soussi A, Ferjani R, Marasco R, Guesmi A, Cherif H, Rolli E, Mapelli F, Ouzari HI, Daffonchio D, Cherif A. 2016. Plant-associated microbiomes in arid lands: diversity, ecology and biotechnological potential. Plant Soil 405:357–370. doi:10.1007/s11104-015-2650-y

[54] Brown SP, Grillo MA, Podowski JC, Heath KD. 2020. Soil origin and plant genotype structure distinct microbiome compartments in the model legume Medicago truncatula. Microbiome 8:139. doi:10.1186/s40168-020-00915-9 32988416 PMC7523075

[55] David AS, Seabloom EW, May G. 2016. Plant host species and geographic distance affect the structure of aboveground fungal symbiont communities, and environmental filtering affects belowground communities in a coastal dune ecosystem. Microb Ecol 71:912–926. doi:10.1007/s00248-015-0712-6 26626912

[56] Hough M, McClure A, Bolduc B, Dorrepaal E, Saleska S, Klepac-Ceraj V, Rich V. 2020. Biotic and environmental drivers of plant microbiomes across a permafrost thaw gradient. Front Microbiol 11:796. doi:10.3389/fmicb.2020.00796 32499761 PMC7243355

[57] Negre Rodríguez M, Pioppi A, Kovács ÁT. 2025. The role of plant host genetics in shaping the composition and functionality of rhizosphere microbiomes. mSystems 10:e0004124. doi:10.1128/msystems.00041-24 40643236 PMC12363198

[58] Otlewska A, Migliore M, Dybka-Stępień K, Manfredini A, Struszczyk-Świta K, Napoli R, Białkowska A, Canfora L, Pinzari F. 2020. When salt meddles between plant, soil, and microorganisms. Front Plant Sci 11:553087. doi:10.3389/fpls.2020.553087 33042180 PMC7525065

[59] Shi X, Zhao X, Ren J, Dong J, Zhang H, Dong Q, Jiang C, Zhong C, Zhou Y, Yu H. 2021. Influence of peanut, sorghum, and soil salinity on microbial community composition in interspecific interaction zone. Front Microbiol 12:678250. doi:10.3389/fmicb.2021.678250 34108953 PMC8180576

[60] Li Y, Qu N, Li S, Zhou H, Yue M. 2025. Ecological mechanisms of microbial assembly in clonal plant Glechoma longituba: from soil to endosphere. Appl Environ Microbiol 91. doi:10.1128/aem.00336-25 PMC12175508 40353652

[61] Harding T, Brown MW, Simpson AGB, Roger AJ. 2016. Osmoadaptative strategy and its molecular signature in obligately halophilic heterotrophic protists. Genome Biol Evol 8:2241–2258. doi:10.1093/gbe/evw152 27412608 PMC4987115

[62] Li Yan, Gu M, Xu W, Zhu J, Chu M, Tang Q, Yi Y, Zhang L, Li P, Zhang Y, Ghenijan O, Zhang Z, Li N. 2025. Whole-genome analysis of Halomonas sp. H5 revealed multiple functional genes relevant to tomato growth promotion, plant salt tolerance, and rhizosphere soil microecology regulation. Microorganisms 13:1781. doi:10.3390/microorganisms13081781 40871285 PMC12388113

[63] Byappanahalli MN, Nevers MB, Korajkic A, Staley ZR, Harwood VJ. 2012. Enterococci in the environment. Microbiol Mol Biol Rev 76:685–706. doi:10.1128/MMBR.00023-12 23204362 PMC3510518

[64] van Overbeek L, Duhamel M, Aanstoot S, van der Plas CL, Nijhuis E, Poleij L, Russ L, van der Zouwen P, Andreo-Jimenez B. 2021. Transmission of Escherichia coli from manure to root zones of field-grown lettuce and leek plants. Microorganisms 9:2289. doi:10.3390/microorganisms9112289 34835415 PMC8622635

[65] Verheijen F, Jelinčić A, Jeffery S, Domingos T, Khodaparast Z, Bastos AC. 2025. Escherichia coli thrives in soil 24 months after grazing exclusion in a rainfed Mediterranean biodiverse pasture. Front Environ Sci 13:2025. doi:10.3389/fenvs.2025.1708183

[66] Luo J, Zhang Z, Hou Y, Diao F, Hao B, Bao Z, Wang L, Guo W. 2021. Exploring microbial resource of different rhizocompartments of dominant plants along the salinity gradient around the hypersaline lake Ejinur. Front Microbiol 12:698479. doi:10.3389/fmicb.2021.698479 34322109 PMC8312270

[67] Halo BA, Aljabri YAS, Yaish MW. 2025. Drought-induced microbial dynamics in cowpea rhizosphere: exploring bacterial diversity and bioinoculant prospects. PLoS One 20:e0320197. doi:10.1371/journal.pone.0320197 40132013 PMC11936235

[68] Danilova IV, Vasileva IA, Gilmutdinova AI, Dyadkina IV, Khusnullina LK, Khasanov DI, Rudakova NL, Sharipova MR. 2023. Characterization of Bacillus pumilus strains with targeted gene editing for antimicrobial peptides and sporulation factor. Microorganisms 11:1508. doi:10.3390/microorganisms11061508 37375011 PMC10303315

[69] Abdelfadil MR, Patz S, Kolb S, Ruppel S. 2024. Unveiling the influence of salinity on bacterial microbiome assembly of halophytes and crops. Environ Microbiome 19:49. doi:10.1186/s40793-024-00592-3 39026296 PMC11256479

[70] Kearl J, McNary C, Lowman JS, Mei C, Aanderud ZT, Smith ST, West J, Colton E, Hamson M, Nielsen BL. 2019. Salt-tolerant halophyte rhizosphere bacteria stimulate growth of alfalfa in salty soil. Front Microbiol 10:1849. doi:10.3389/fmicb.2019.01849 31474952 PMC6702273

